# A Case of Red Granular Kidney

**Published:** 1908-01-11

**Authors:** 


### A CASE OF RED GRANULAR KIDNEY.

It has long been the teaching of clinical physicians that "in cases of red granular kidney hæmorrhage may occur anywhere in the body," but it is not, perhaps, fully realised that severe bleeding from the kidney is one of these possible hæmorrhages. One remembers epistaxis and cerebral hæmorrhage easily enough, but one is perhaps a little apt to forget that the brain and the nose are not the only places in which the arterioles can give way when the blood pressure is high. It sometimes happens that the vessel which leaks is in the kidney itself. The hæmaturia has then a very different pathology from that of acute nephritis. There may be no active inflammation in the kidney at all. The bleeding may be so severe that growth of the kidney, papilloma, carcinoma, or sarcoma, is suspected and even operated for.

It may be mentioned that similar hæmorrhage from the renal vessels is a conspicuous symptom in cases of cystic kidney; this is noteworthy, for although cystic kidney disease is often regarded as rare, it is sufficiently common for one general practitioner to have had three cases of it within six months, all verified by kidney examination at operation or autopsy, and all giving profuse hæmaturia as the first and most prominent symptom.

The following is an example of a patient with a granular kidney of which extreme hæmaturia was the only symptom. The case is published *in extenso* in the Johns Hopkins Hospital Reports by H. A. Fowler, Esq., M.D.

A carpenter, aged 60, was first seen on February 1, 1904. He complained of passing bloody urine, and he had suffered from attacks of severe hæmaturia for the past four years.

The trouble began as follows: four years ago, on getting up one night as usual to pass his water he noticed that the urine was bloody. There was no pain associated with micturition, and no other urinary disturbance. Since that time the urine has been constantly bloody, at times more so than others. He had no other urinary troubles, except that one year ago he had one attack of severe pain in the loins, more severe on the right side; the pain did not radiate; it was so severe that morphia had to be given. Once or twice he has had pains which suggested something obstructive, possibly blood clot, passing down the right ureter. He has taken various kinds of medicine, and has been treated by rest in bed and by electricity, without, however, experiencing any diminution in the amount of blood in the urine.

He has been strictly moderate as regards alcohol. At the period of the onset of the hæmaturia he was working at his trade, and he has continued at work until recently. The hæmaturia has gradually sapped his strength so that now he is easily tired and does not feel equal to his work. He has not wasted. He has had no œdema of feet or legs, and no respiratory difficulty.

The pulse rate was 56, and the pulse itself of full volume and increased tension. There was no obvious abnormality detected about the heart. Neither kidney could be palpated, and there was no pain on palpating firmly over kidneys and ureters.

The urine was equally bloody throughout micturition, very dark red, acid, with a specific gravity of 1,025. Microscopical examination showed red blood corpuscles in abundance, with a corresponding number of leucocytes and albumen; no renal tube casts could be found, notwithstanding repeated search, nor were crystals, particles of new growth, nor any pus cells to be found. Tubercle bacilli were looked for with negative result. The quantity passed in 24 hours averaged 40 oz.

No effort was spared to try and determine the cause of the constant hæmorrhage. The bladder was examined carefully; no residual urine was present; cystoscopic examination was carried out with some difficulty owing to the presence of so much blood, but it was ascertained with certainty that the prostate was not interfering with the urethra, that the bladder mucosa was free from ulceration and from growth, that the left ureter was discharging clear urine, and that the right ureter was constantly emitting blood.

It was clear, therefore, that the condition was one of hæmorrhage from one kidney only; and very naturally a small tumour was suspected in the right renal pelvis. It seemed clear that operation was indicated, because the constant hæmorrhage was weakening the patient so much. However, at the patient's own desire it was postponed, and he went back and tried to go on with his work. In August, 1904, however, he had to give up, and come back for operation. The hæmaturia had been going on all the time and there was no fresh symptom and no change in the physical signs; the urine was as before.

The primary object of the operation was to check the hæmorrhage; the kidney was cut down upon, therefore; it contained no stone or other removable body, so that nephrectomy was done.

The patient got well rapidly after the operation, and went back to his work.

He now passes about 40 oz. of urine in 24 hours, pale in colour, and of specific gravity varying from 1,012 to 1,020, with the faintest trace of albumen, a few hyaline tube casts, and a very small number of red blood discs.

### Description of the Kidney Removed.

It was a regular red granular kidney. Microscopically it showed the usual increase of connective tissue, fibroid obliteration of many of the glomeruli, with contraction and indentation of the cortex. Some of the tubules contained hyaline casts, and there were many blood extravasations in the renal tissue. The condition was one of diffuse and chronic interstitial nephritis.

In view of the present condition of the urine, the left kidney is almost certainly in a similar condition, but without the same tendency of the vessels in it to give way.

